# Phase I study of temozolomide plus paclitaxel in patients with advanced malignant melanoma and associated *in vitro* investigations

**DOI:** 10.1038/sj.bjc.6602438

**Published:** 2005-03-08

**Authors:** A Azzabi, A N Hughes, P M Calvert, E R Plummer, R Todd, M J Griffin, M J Lind, A Maraveyas, C Kelly, K Fishwick, A H Calvert, A V Boddy

**Affiliations:** 1 Northern Institute for Cancer Research, University of Newcastle, Newcastle upon Tyne NE2 4HH, UK; 2 Northern Centre for Cancer Treatment, Newcastle General Hospital, Newcastle upon Tyne, UK; 3 Academic Department of Oncology, The Princess Royal Hospital, Hull, UK

**Keywords:** combination, pharmacokinetics, interaction, epothilone B

## Abstract

The purpose of this study was to determine activity of temozolomide combined with paclitaxel or epothilone B *in vitro*, and to investigate the combination of temozolomide with paclitaxel in a Phase I clinical trial. Melanoma cell lines A375P and DX3 were treated with temozolomide and either paclitaxel or epothilone B. Combination indices were determined to assess the degree of synergism. In a clinical study, 21 patients with malignant melanoma were treated with increasing doses of temozolomide (orally, days 1–5), in combination with a fixed dose of paclitaxel (i.v. infusion day 1), followed by dose escalation of the latter drug. Cycles of treatment were repeated every 3 weeks. Pharmacokinetics of both agents were determined on day 1, with temozolomide pharmacokinetics also assessed on day 5. All three compounds were active against the melanoma cell lines, with epothilone B being the most potent. There was a strong degree of synergism between temozolomide and either paclitaxel or epothilone B. In the clinical study, no pharmacokinetic interaction was observed between temozolomide and paclitaxel. Dose escalation of both drugs to clinically active doses was possible, with no dose-limiting toxicities observed at 200 mg m^−2^ day^−1^ temozolomide and 225 mg m^−2^ day^−1^ paclitaxel. There were two partial responses out of 15 evaluable patients. One patient remains alive and symptom-free at 4 years after treatment. Temozolomide and paclitaxel may be administered safely at clinically effective doses. Further evaluation of these combinations in melanoma is warranted.

Stage IV melanoma carries a very poor prognosis (Balch *et al*, 2001) and a median survival of no more than 6–9 months. The 5-year survival is at best 5–10% (Lotze *et al*, 2001). Several drugs have been used in the treatment of stage IV melanoma, including dacarbazine (DTIC), nitrosoureas, platinum compounds and vinca alkaloids, with only modest success.

Paclitaxel (Taxol™) is a member of the taxane group of antitumour agents. It was discovered in the early sixties by the National Cancer Institute (Rowinsky and Donehower, 1995). Early clinical studies indicated activity against a number of tumours including ovarian, breast, lung and melanoma (Rowinsky *et al*, 1990; Einzig *et al*, 1991b; Ettinger, 1993; Martin, 1993). Paclitaxel was first approved by the FDA in 1992 to be used as a second-line treatment for ovarian carcinoma (Arbuck *et al*, 1993). Against metastatic malignant melanoma, paclitaxel has a response rate of 18–20% (Legha *et al*, 1990; Einzig *et al*, 1991a; Wiernik and Einzig, 1993). Paclitaxel exerts its antitumour activity by binding to the *β* subunit of tubulin (Horwitz, 1994) and stabilising microtubules. The mechanisms by which cells become resistant to paclitaxel are thought to be either due to mutations in tubulin or to overexpression of drug efflux pumps such as MDR1. The main toxicities associated with paclitaxel are: hypersensitivity reactions, neutropaenia and peripheral neuropathy, usually sensory.

Temozolomide (Temodal™) is an oral alkylating agent, related to DTIC. After administration, temozolomide spontaneously decomposes to 5-(3-methyltriazen-l-yl)imidazole-4-carboxamide (MTIC), which then yields AIC and methyl diazonium (Denny *et al*, 1994). The latter alkylates DNA, primarily at the *O*^6^ of guanine. Bioavailability of temozolomide is nearly 100% when administered orally, with good distribution to tissue (Baker *et al*, 1999; Brada *et al*, 1999). Activity has been demonstrated against a number of tumours (Bleehen *et al*, 1995; Dhodapkar *et al*, 1997; Middleton *et al*, 2000b). In malignant melanoma, activity was comparable to that of dacarbazine (Middleton *et al*, 2000a, 2000b).

Cancer cells may acquire resistance to temozolomide by a number of mechanisms, including overexpression of *O*^6^-alkylguanine-DNA alkyltransferase (Lee *et al*, 1994; Middleton *et al*, 1998), deficiency or mutation in the mismatch repair (MMR) pathway (D'Atri *et al*, 1998) and increased expression of proteins that inhibit apoptosis, such as Bcl-2 (Selzer *et al*, 1998). Temozolomide is well tolerated (Newlands *et al*, 1992; O'Reilly *et al*, 1993; Bleehen *et al*, 1995; Dhodapkar *et al*, 1997), with thrombocytopaenia as the main toxicity. This is usually self-limiting and most patients recover by day 28.

Individually, paclitaxel and temozolomide have modest activity against metastatic malignant melanoma. As they have different mechanisms of action, nonoverlapping toxicities and differing mechanisms of resistance, the combination of paclitaxel and temozolomide has been investigated in a Phase I study in melanoma patients. In addition to the clinical trial, *in vitro* growth inhibition studies were performed with the combination of temozolomide and either paclitaxel or epothilone B, a novel antimicrotubule compound (Rothermel *et al*, 2003).

## METHODS

### *In vitro* studies

Two melanoma cell lines, A375P and DX3, were cultured using RPMI 1640 culture medium with L-glutamine supplemented with 10% foetal calf serum and penicillin/streptomycin in 5% CO_2_ incubators at 37°C. Cells were exposed to varying concentrations of temozolomide, paclitaxel or epothilone B individually for three doubling times and IC_50_ values determined in triplicate using the SRB assay.

Drug combination studies were conducted on each cell line using either temozolomide plus paclitaxel or temozolomide plus epothilone B. In each case, cells were exposed to the two agents simultaneously. Drug concentrations used in combination were at 3, 1, 0.3, 0.1, 0.01 times the IC_50_ concentration for each drug. Data were analysed using the Calcusyn software and the method of Chou and Hayball (1996). The combination index (CI) for each drug combination was calculated, where CI=1 denotes an additive effect, CI<1 indicates synergy and CI>1 suggests antagonism of effect.

### Clinical study

The primary objectives of the study were to determine the maximum tolerated dose of the combination of paclitaxel and temozolomide, and to identify the haematological and nonhaematological toxicities of the combination. Secondary objectives were to determine the response rate and response duration from the combination, and to determine the pharmacokinetics of temozolomide and paclitaxel when given in combination.

Adult patients with a diagnosis of stage IV melanoma were included. All were chemotherapy naïve, with no clinical evidence of CNS metastases, WHO performance status of 0–2, a life expectancy of at least 12 weeks and adequate haematological, renal and hepatic function. Patients had at least one measurable lesion. All patients signed an informed consent form prior to participating in the trial, which was approved by the ethics committee of the Newcastle Hospitals Trust and of the Medical School, University of Kingston upon Hull. All patients had prestudy biochemical, clinical and radiological assessments before receiving the first cycle of chemotherapy and subsequently during the study. Toxicities were assessed using the Common Toxicity Criteria (Version 2.0).

**Figure 1:**
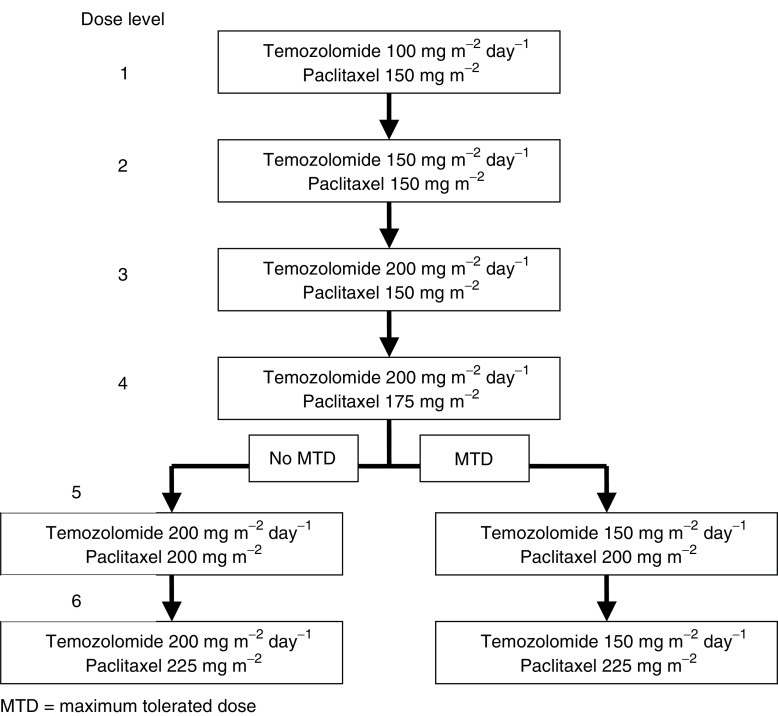
Dose escalation scheme, indicating daily dose of temozolomide, given on each of 5 consecutive days. Dose of paclitaxel administered on day 1. The dose levels used in the current study are indicated by number. Dose level 4 represents full therapeutic doses of each agent. After dose level 4, subsequent dose changes depended on the occurrence of dose-limiting toxicities to define the maximum tolerated dose (MTD).

Dose levels for the study were predefined as in Figure 1; however, intermediate dose levels were allowed. Paclitaxel was administered intravenously as a 3-h infusion on day one of each cycle. Standard premedications were used to prevent hypersensitivity reactions. Temozolomide was given orally on the morning of days 1–5 at least 1 h before or 2 h after breakfast. Post chemotherapy, emesis was treated prophylactically, with ondansetron or metoclopramide orally.

On day 1 of cycle 1, blood samples (5 ml) for analysis of temozolomide were collected in pre-chilled heparinised tubes at 10, 20, 30, 60, 90, 120, 150, 180, 240, 360 and 1320 min after administration of temozolomide, plasma was separated and 2 ml was transferred to a plastic tube containing 0.1 ml of 8.5% phosphoric acid. For analysis of paclitaxel, 5 ml heparinised blood samples were taken pretreatment and at 90, 180, 185, 195, 210, 225, 240, 270, 300, 360, 420, 540, 1260 and 1620 min from the start of infusion. Sampling for temozolomide pharmacokinetics was repeated on day 5 of cycle 1. Determinations of concentrations of paclitaxel or temozolomide in plasma were performed by HPLC, as previously described (Siddiqui *et al*, 1997; Estlin *et al*, 1998).

## RESULTS

### *In vitro* studies

Against the two melanoma cell lines, paclitaxel and temozolomide had very different activity. Values for IC_50_ (mean±s.d.) for temozolomide (835±110 and 821±75 *μ*M) and paclitaxel (15.3±2.5 nM and 13.5±1.5nM) were similar for the DX3 and A375P cell lines. The cell lines were 30- to 40-fold more sensitive to epothilone B, with IC_50_ values of 0.55±0.06 and 0.31±0.05 nM against DX3 and A375P cells, respectively.

For the DX3 cell line, the combination of temozolomide with paclitaxel produced CI values ranging from 0.02 at a fractional effect (FE) of 0.65 to a CI of 0.4 at an FE of 0.95. Corresponding values for the DX3 cells with temozolomide and epothilone B were CI=0.01 (FE=0.84) and CI=0.55 (FE=0.96). In the A375P cell line, temozolomide plus paclitaxel produced a CI of 0.15 at an FE of 0.1 and 0.66 at an FE of 0.9. Corresponding values for the combination with epothilone in the A375P cells were CI=0.07 (FE=0.05) and CI=0.79 (FE=0.98). Taken together, these data suggest synergism of action of these drug combinations against these two melanoma cell lines.

### Clinical study

A total of 22 patients (13 male and nine female subjects) with a median age of 52.5 years (range 29–69 years) were recruited into the clinical study (Table 1). Patients received a total of 61 cycles of the combination chemotherapy, with an average of 2.8 cycles per patient. A total of 17 patients were assessable for toxicities and 15 patients were assessable for disease response. Six dose levels were explored (Figure 1).

Four patients were withdrawn from the study due to development of brain metastases within 28 days of enrolment. One patient was consented and did not receive treatment due to bleeding skin lesions and one patient progressed clinically and was taken off study after 22 days. One patient with apparently recurrent disease was found, on review of CT scans after six cycles of treatment, to have simple liver cysts.

Of the 17 patients assessable for toxicity, two experienced neutropaenia Grade 3 (Table 2). There was one episode of Grade 4 neutropaenia in a patient who also had Grade 3 thrombocytopaenia and anaemia. There were also two other cases of Grade 3 thrombocytopaenia. Patients 5 and 10 required a 50% dose reduction in temozolomide due to hematological toxicity, with patient 11 requiring a 50% dose reduction in both drugs. Nonhematological toxicities included arthralgia, nausea and vaginal thrush. Two patients experienced an allergic reaction to Taxol, but in only one of these did this limit treatment.

Of the 15 patients assessable for response, there were two partial responses. Patient 1, who had primary disease in the gall bladder with liver secondaries, showed a sustained response with no evidence of progressive disease after nine cycles. At 4 years after starting the trial, the patient remains well with only minimal evidence of liver disease. Patient 206, whose primary site was the skin of the left shoulder, with some deposits in the left axiliary lymph node, was treated initially on dose level 6. She had a partial response after two cycles, which was sustained on CT scan after a further four cycles of treatment. This patient subsequently suffered a recurrence of disease 3 months after the end of treatment, received radiotherapy and further chemotherapy including single agent temozolomide, but died of disease progression nearly 2 years after entry into this trial. In addition, patient 5 at dose level 2 and patient 10 at dose level 4 had mixed or minor responses. Patient 204 had stable disease up to course 4, but was withdrawn from study due to brain metastases.

### Pharmacokinetic studies

**Figure 2:**
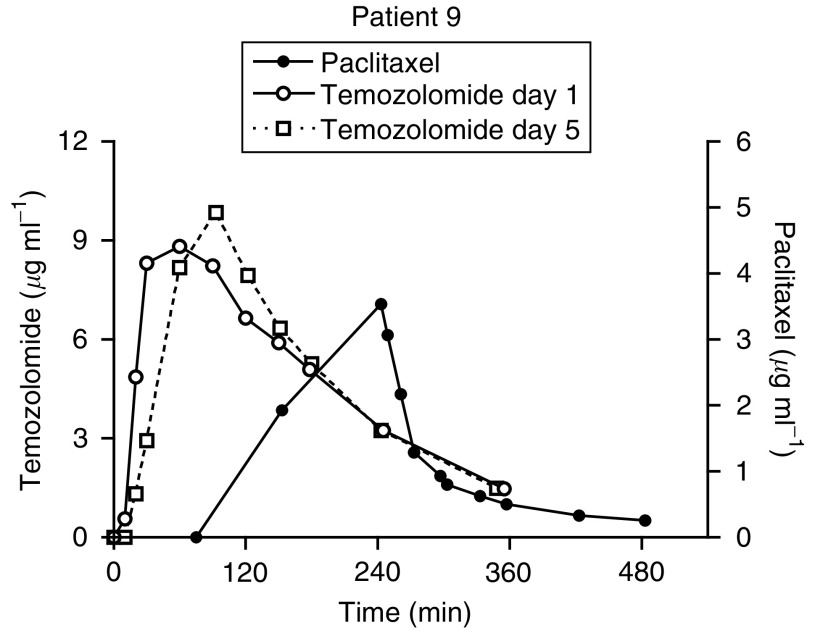
Time course of temozolomide (left axis) and paclitaxel (right axis) plasma concentrations on day 1 of therapy. The oral dose of temozolomide was administered first, with the 3 h infusion of paclitaxel started 60 min later. Data from day 5 administration of temozolomide are superimposed for comparison.

Temozolomide was administered at three different doses (100, 150 and 200 mg m^−2^ day^−1^). Although four patients were treated with 150 mg m^−2^ day^−1^ temozolomide (combined with 150 mg m^−2^ paclitaxel), reliable pharmacokinetic data for temozolomide were available from only one patient. One patient on dose level 3 at 200 mg m^−2^ day^−1^ had anomalous data, with increasing plasma concentrations throughout the sampling period. A representative graph of temozolomide and paclitaxel plasma concentrations in a patient treated at dose level 3 is shown in Figure 2.

There was no significant difference among the estimates of half-life, Cl/F or Vz/F for temozolomide at these different doses of temozolomide (Table 3), with an apparent dose-linear increase in AUC comparing doses between 100 and 200 mg m^−2^ (Table 3). The different doses of paclitaxel (150, 175, 200 and 225 mg m^−2^) administered in conjunction with the highest dose of temozolomide (200 mg m^−2^) did not appear to influence the pharmacokinetics of the latter drug (data not shown). Comparing the pharmacokinetics of temozolomide on day 1 (with paclitaxel) and day 5 (without paclitaxel), there was no difference in *C*_max_ or AUC between the 2 days of study (Table 3).

A summary of the pharmacokinetic data for paclitaxel at each of four dose levels is given in Table 4. Clearance of paclitaxel was greater at the lowest dose level of 150 mg m^−2^ (608±211 ml min^−1^, all patients dose levels 1–3), compared to that observed at 175–225 mg m^−2^ (373±100 to 363±120 ml min^−1^). For dose levels 1–3, where the paclitaxel dose was held constant, but the dose of temozolomide varied from 100 to 200 mg m^−2^ day^−1^, there appears to be an increase in paclitaxel clearance as the dose of temozolomide was escalated (Table 4). However, the numbers of patients at each dose level are small and one-way analysis of variance on the log-transformed data indicated no significant effect. Comparison of paclitaxel clearance from patients in the current study with those from previously published studies suggests that those patients at dose level 1 have an unusually low clearance, while clearance values in patients at dose level 3 are larger than expected (see below).

## DISCUSSION

This investigation combines *in vitro* studies of temozolomide and tubulin binding drugs with a Phase I clinical study of temozolomide and paclitaxel in melanoma. Interest in the use of these two agents in melanoma has been stimulated by their single-agent activities (Wiernik and Einzig, 1993) and nonoverlapping toxicities and mechanisms of resistance.

*In vitro* studies in two melanoma cell lines showed sensitivity to temozolomide and paclitaxel in line with reported IC_50_ values for a variety of other cell lines. Sensitivity to temozolomide is reported to depend on the activity of the repair enzyme *O*^6^-alklyguanine alkyltransferase (ATase) and on mismatch repair (MMR) function (Pepponi *et al*, 2003). While the MMR status of these cell lines is not known, the relatively high IC_50_, coupled with the low ATase activity of the A375P cell line (95 fmol mg^−1^ protein) (Wedge *et al*, 1997), may suggest a deficiency in MMR. Many tumour cells have only a moderate sensitivity to temozolomide (IC_50_ around 10–1000 *μ*M) (Taverna *et al*, 2000; Pepponi *et al*, 2003), with a greater degree of sensitivity to paclitaxel (IC_50_ 1–10nM) (Rowinsky and Donehower, 1995; Lamendola *et al*, 2003). Resistance of melanoma cell lines to temozolomide is associated with deficiencies in mismatch repair and/or high activity of the repair enzyme *O*^6^-alkylguanine-DNA alkyltransferase (Pepponi *et al*, 2003). Interestingly, these two melanoma cell lines were also sensitive to epothilone B (IC_50_ 0.3 and 0.5 nM). In a Phase I study of epothilone B, plasma concentrations exceeded 1 nM for approximately 8 h (Rothermel *et al*, 2003).

The combination studies with temozolomide and either paclitaxel or epothilone B showed strong signs of synergy. At the higher dose combinations used, effectively all of the cells were killed. The mechanism of such synergy may relate to the mitotic block following paclitaxel or epothilone B treatment. It has been suggested that cells become apoptotic following escape from G2M blockade, and it may be that such cells are then sensitised to the actions of alkylating agents such as temozolomide. Previous studies have indicated additive effects of alkylating agents with paclitaxel (Liebmann *et al*, 1994). Combinations of temozolomide with tubulin binders such as the agents studied here have not been reported previously and may show useful activity in other tumour types. Although only two melanoma cell lines were studied, the activity of these agents in combination, at clinically relevant concentrations, indicates that further evaluation is warranted.

The clinical study, with a dose escalation of temozolomide followed by escalation of paclitaxel, showed that in combination both drugs could be given at full therapeutic doses. Indeed, the toxicity experienced was relatively mild even at the highest dose of paclitaxel (225 mg m^−2^) permitted by the protocol. Although grade 3 and 4 toxicities were observed at dose level 4 (200 mg m^−2^ day^−1^ temozolomide + 175 mg m^−2^ paclitaxel), none occurred at the subsequent higher dose levels. There was some evidence of cumulative toxicity, with patient 10 at dose level 4 withdrawn from treatment due to Grade 4 thrombocytopaenia after course 4. Further dose escalation may be possible, but the clinical benefit of this is unknown. A number of patients gained some clinical benefit from treatment with temozolomide and paclitaxel. Patient 1 showed a good response and went on to receive nine courses of treatment, remaining well on long-term follow-up. Patient 10 had a minor response and patient 206 had a partial response after course 3, receiving six courses in total. Patient 7 received six courses of treatment for what appeared to be a recurrence of liver metastases. However, liver scans revealed the presence of benign cysts. In this Phase I study, the primary aim was to determine the safety of temozolomide and paclitaxel in combination. Nevertheless, the level of activity observed, over a range of dose levels, is promising.

Other clinical studies of a combination of a taxane with monofunctional alkylating agent have been reported. Paclitaxel has been given at doses up to 250 mg m^−2^ with DTIC up to 1000 mg m^−2^. This combination was well tolerated, but did not show a higher response rate than either agent used alone (Feun *et al*, 2002). Similarly, docetaxel has been combined with DTIC, but again the response rate was not dissimilar to single-agent therapy (Bafaloukos *et al*, 2002a). The combination of temozolomide with docetaxel has recently been reported to be safe and to have good activity in a Phase II study (Bafaloukos *et al*, 2002b). Dacarbazine may not be a good drug for use in these combinations as it requires metabolic activation, which may be inhibited by coadministered drugs such as the taxanes.

Recent clinical studies in malignant melanoma have explored other schedules and combinations of these two drugs. Temozolomide has been combined with thalidomide (Hwu *et al*, 2003) or with interferon-*α*, using schedules similar to that used here, or with daily or three-times daily administration for 6 weeks out of eight. Most promising results have been obtained in combination with thalidomide (Danson *et al*, 2003), but that combination requires further investigation. Paclitaxel has been investigated on a weekly schedule (Zimpfer-Rechner *et al*, 2003) or every 4 days for three doses in a three week cycle. The latter schedule produced a 15% response rate (Bedikian *et al*, 2004). Combinations with carboplatin have also been investigated, but with no therapeutic benefit (Zimpfer-Rechner *et al*, 2003). Given the results presented here, exploration of other schedules for both drugs may be beneficial.

The combination of temozolomide with paclitaxel involves the administration of one drug orally, while the other is given by a continuous 3 h infusion. The relative timings of the two drugs may be governed by clinical concerns, such as withholding the oral drug in case a hypersensitivity reaction is seen to the intravenous one. Hypersensitivity reactions were seen in only two patients in this study. Regardless of the order of administration, there is no *a priori* reason to suspect a pharmacokinetic interaction between temozolomide and paclitaxel. One is eliminated almost entirely by a spontaneous chemical reaction, while the other is subject to metabolism, mediated by CYP450 enzymes, and to biliary excretion.

The pharmacokinetic parameters derived for temozolomide in combination with paclitaxel were very similar to those reported previously for temozolomide alone (Jen *et al*, 2000). Also, there was no evidence of nonlinearity of pharmacokinetics as the dose of temozolomide was increased and no effect of increasing the concurrent dose of paclitaxel from 150 to 225 mg m^−2^. Although at the lowest dose level of paclitaxel (150 mg m^−2^) there did appear to be an increase in clearance with increasing dose of temozolomide (100–200 mg m^−2^ day^−1^), this was not statistically significant. Previous studies with paclitaxel at a dose of 150 mg m^−2^ indicated a clearance of 588±31 ml min^−1^ (Siddiqui *et al*, 1997), almost identical to that reported at dose level 2. At dose level 3, the two patients with full paclitaxel data had unusually high clearances, but the third patient, in whom data were available only to 8 h, had an approximate clearance value nearer to that expected. There was no evidence of increased clearance of paclitaxel in combination with the same dose of temozolomide at dose level 4. The expected phenomenon of apparent decrease in clearance with increasing dose of paclitaxel was observed, which may cloud the interpretation of any apparent interaction. This apparent nonlinearity in pharmacokinetics is now known to be due to the action of the solubilising agent Cremophor, which sequesters paclitaxel in a dose-dependent manner (van Zuylen *et al*, 2001). In conclusion, in the absence of a within patient crossover study, there appears to be no pharmacokinetic interaction between temozolomide and paclitaxel when given in combination.

The *in vitro* data presented here indicate that the combination of temozolomide with the microtubule agent epothilone B or paclitaxel produces a synergistic inhibition of tumour cell growth. The clinical study of the combination of temozolomide with paclitaxel shows that this combination is well tolerated, not complicated by any pharmacokinetic interaction and has modest activity in a Phase I setting. Although the response rate in this Phase I study was not dissimilar to that of the individual agents, the combination of the two drugs at full therapeutic doses could be investigated further. Combinations of temozolomide with epothilone B may also be beneficial, but require initial clinical evaluation.

## Figures and Tables

**Table 1:** Patient summary

**Patient**	**Dose level**	**Gender**	**Age (years)**	**Clinical outcome**
1	1	F	51	Excellent response to treatment, had nine cycles
2	1	F	62	PD after C2
3	1	F	57	PD after C4
4	2	M	31	Withdrawn due to brain metastases
5	2	F	41	Improvement in CT after C3, but PD after C5
6	2	M	55	PD after 22 days
7	2	M	37	After C6, MRI revealed cysts and no cancer
8	3	M	55	PD after 31 days
9	3	M	52	PD after C2
10	4	F	50	Minor response after C3. Withdrawn due to G4 thrombocytopenia after C4
11	4	F	52	Stopped after C2 due to haematoxocity and allergic reaction to taxol
12	4	F	66	PD after C3
201	4	M	65	PD after C2
13	4	F	38	Withdrawn due to brain metastases after C3
14	4	M	29	Not given treatment, bleeding lesion
202	4	M	53	PD after C2
203	5	M	68	Withdrawn due to brain metastases after C1
204	5	M	63	SD after C2 and C4, but withdrawn due to brain metastases after C4
205	5	M	54	Withdrawn due to brain metastases after C1
206	6	F	52	PR after C3, had six cycles in total
20	6	M	41	PD after C2
21	6	M	69	PD after C2

Patients were recruited at two centres, with different patient number schemes at each center.

PD=progressive disease; C2=course 2, etc.; CT=computerised tomography scan; MRI=magnetic resonance imaging scan; G4=Grade 4, etc., M=male; F=female; PR=partial response; SD=stable disease.

**Table 2:** Haematological and nonhaematological toxicity by dose level

**Dose level**	**No. of patients**	**No. of cycles**	**Haematological toxicities**	**Nonhaem. toxicities**	**Best responses**
1	3	15	G2 neutropaenia	G1 Arthralagia	1 PR
2	4	12	G3 anaemia	Allergic reaction to taxol	1 MR
			G3 thrombocytopaenia		
3	2	3	G3 neutropaenia	G2 Arth.	
4	6	16	G4 neutropaenia × 1; G3 neutropaenia × 1	G2 Arth.	1 MR
			G3 thrombocytopaenia × 2; G3 anaemia × 1	Allergy to taxol	
5	3	5	G2 neutropaenia		1 SD
6	3	10	G2 neutropaenia × 2		1 PR

PR=partial response; MR=mixed response; SD=stable disease.

**Table 3:** Temozolomide pharmacokinetic parameters

**Dose level**	**Half-life (min)**	**Clearance/*F* (ml min^−1^)**	**Volume of distribution/*F* (l)**	**AUC day 1 (*μ*g ml^−1^ min^−1^)**	**AUC day 5 (*μ*g ml^−1^ min^−1^)**
1 (*n*=3) (100 mg m^−2^ day^−1^)	98±20	157±10	22.2±5.9	1064±3	921^a^
2 (*n*=1) (150 mg m^−2^ day^−1^)	241	157	54	1501	
3–6 (*n*=14) (200 mg m^−2^ day^−1^)	122±55	188±55	30.5±5.5	2106±608	1946±580 (*n*=11)

*F* indicates bioavailability, which is not determined here.

AUC is the area under the plasma concentration–time curve.

a Data available on only one patient at this dose level.

One patient at 200 mg m^−2^ day^−1^ had anomalous data, with increasing plasma concentrations throughout the sampling period.

**Table 4:** Paclitaxel pharmacokinetic parameters

**Dose level**	**Half-life (min)**	**Clearance (ml min^−1^)**	**Volume of distribution (litres)**	**AUC (*μ*g ml^−1^ min^−1^)**
1 (150 mg m^−2^)	470, 522	364, 430	247, 324	632, 600
2 (150 mg m^−2^)	502, 383	652, 513	473, 283	359, 480
3 (150 mg m^−2^)	550, 412, 105^a^	779, 909, 505^a^	619, 540, 76^a^	385, 300, 523^a^
4 (175 mg m^−2^)	410±74	373±100	216±53	858±255
5 (200 mg m^−2^)	495, 198^a^	320, 717^a^	228, 205^a^	1162, 544^a^
6 (225 mg m^−2^)	298±87	363±120	152±63	1220±416

a Data only to 8 h.
